# Lysophosphatidic Acid Receptor 3 Promotes Mitochondrial Homeostasis against Oxidative Stress: Potential Therapeutic Approaches for Hutchinson–Gilford Progeria Syndrome

**DOI:** 10.3390/antiox11020351

**Published:** 2022-02-10

**Authors:** Jui-Chung Chiang, Wei-Min Chen, Ciara Newman, Benjamin P. C. Chen, Hsinyu Lee

**Affiliations:** 1 Department of Radiation Oncology, University of Texas Southwestern Medical Center, Dallas, TX 75390, USA; Jui-Chung.Chiang@UTsouthwestern.edu (J.-C.C.); Wei-Min.Chen@UTSouthwestern.edu (W.-M.C.); Ciara.Newman@UTSouthwestern.edu (C.N.); 2 Department of Life Science, National Taiwan University, Taipei 10617, Taiwan

**Keywords:** lysophosphatidic acid, mitochondrial homeostasis, oxidative stress, Hutchinson-Gilford progeria syndrome

## Abstract

Lysophosphatidic acid (LPA) is a growth factor-like lipid mediator that regulates various physiological functions via activation of multiple LPA G protein-coupled receptors. We previously reported that LPA suppresses oxidative stress in premature aging Hutchinson-Gilford progeria syndrome (HGPS) patient fibroblasts via its type 3 receptor (LPA_3_). Mitochondria have been suggested to be the primary origin of oxidative stress via the overproduction of reactive oxygen species (ROS). Mitochondria are responsible for producing ATP through oxidative phosphorylation (OXPHOS) and have a calcium buffering capacity for the cell. Defects in mitochondria will lead to declined antioxidant capacity and cell apoptosis. Therefore, we aim to demonstrate the regulatory role of LPA_3_ in mitochondrial homeostasis. siRNA-mediated depletion of LPA_3_ leads to the depolarization of mitochondrial potential (ΔΨm) and cellular ROS accumulation. In addition, the depletion of LPA_3_ enhances cisplatin-induced cytochrome C releasing. This indicates that LPA_3_ is essential to suppress the mitochondrial apoptosis pathway. LPA_3_ is also shown to improve mitochondrial ADP-ATP exchange by enhancing the protein level of ANT2. On the other hand, LPA_3_ regulates calcium uptake from the ER to mitochondria via the IP3R1-VDAC1 channel. Moreover, activation of LPA_3_ by selective agonist OMPT rescues mitochondrial homeostasis of H_2_O_2_-induced oxidative stress cells and HGPS patient fibroblasts by improving mitochondrial ΔΨm and OXPHOS. In summary, our findings imply that LPA_3_ acts as the gatekeeper for mitochondrial healthiness to maintain cell youth. Furthermore, LPA_3_ can be a promising therapeutic target to prevent mitochondrial oxidative stress in aging and HGPS.

## 1. Introduction

Lysophosphatidic acid (1- or 2-acyl-sn-glycerol 3-phosphate/radyl-glycerol-phosphate, LPA) is a bioactive lipid mediator produced both intracellularly and extracellularly from membrane phospholipids and is detected robustly in all eukaryotic tissues and blood plasma [1]. LPA evokes various cellular responses by activating six distinct G protein-coupled receptors (GPCRs) localized in the plasma membrane of various cells, mediating cell proliferation, migration, and cytoskeletal reorganization [2]. These receptors are subdivided into the endothelial cell differentiation gene family (Edg, LPA_1_-LPA_3_) [3] and the P2Y purinergic receptor family (LPA_4_-LPA_6_) [4]. Upon binding with LPA, conformational changes of LPA receptors allow them to act as guanine nucleotide exchange factors for one or more of the four classes of heterotrimeric G proteins, G_12/13_, G_q/11_, G_i/o_, and G_s_, which initiate signaling cascades through downstream molecules such as Rho, IP3-DAG, adenylyl cyclase and Ras/mitogen-activated protein kinase (MAPK) pathways [5,6,7]. Studies have documented the wide range of LPA downstream signaling pathways involving diverse physiological and pathophysiological functions, including nervous/vascular system development, reproduction, angiogenesis, cancer progression, and hematopoiesis [8,9,10].

Moreover, we also showed that LPA can exert different functions according to its GPCRs expression pattern. For instance, our recent studies suggest that LPA_2_ and LPA_3_ expressed at different stages of hematopoietic progenitors decide the fate of blood cell differentiation [11,12]. In addition, studies proposed the concept that the profile of LPA receptors change during the cell aging process which affect activities of the downstream pathway [13,14]. Similarly, we also observed that LPA_3_ decreases in the senescent and premature aging disease, Hutchinson-Gilford progeria symptom (HGPS) model [11,15]. LPA_3_ knockout zebrafish larvae we established showed senescent phenotypes and lower locator activity, and the adult fish have a shorter life span than wild-type zebrafish [16]. LPA also protects cells from mitochondrial apoptosis by enhancing JNK-mediated phosphorylation of Bcl-2 and ERK1/2 pathways and by accumulating the anti-apoptotic protein, Mcl-1 [17,18]. These beneficial effects of LPA on mitochondria can align with our findings that activation of LPA_3_ in HGPS cells rescued premature aging phenotype. Notably, our recently published studies showed that activation of LPA_3_ stabilizes the protein of nuclear factor erythroid 2-related factor (Nrf2), consequently promoting the expression of antioxidants and reducing mitochondrial superoxide [15]. This evidence suggests that the potentiation of LPA_3_ signaling may constitute a new approach against aging progress and mitochondrial oxidative stress.

Mitochondria are currently considered a critical component of intracellular signaling, immune response, and a modulator of cell replication, more than simple bioenergetic factories. The development of a wide range of aging-related diseases is highly associated with the decline of mitochondrial quality and activity. It is widely accepted that, during the aging process, the free radical (reactive oxygen species, ROS) leads to cumulative damage and irreversible cell damage. The mitochondria are considered the central regulators of redox activities and oxidative stress, called mitochondrial ROS (mtROS). Therefore, mitochondrial dysfunctions have been suggested to be the hallmark and driver of aging [19]. In addition, HGPS, which is severe premature aging, shows similar signaling pathway patterns as normal aging cells [20]. This syndrome is typically caused by a silent mutation (c. 1824C>T; p. Gly608Gly) of LMNA that activates an alternative pre-mRNA cryptic splicing site and causes a 150-nucleotide, 50 amino acid deletion. The missing sequence includes a recognition site for ZMPSTE24 endoprotease, leading to the accumulation of an un-cleaved Lamin A isoform, named progerin [21]. HGPS cells display significant dysregulation of oxidative phosphorylation (OXPHOS), mitochondrial activities, and ROS scavenging [22,23]. Treatments aimed to improve the mitochondrial homeostasis were shown to ameliorate the cell senescence of HGPS cells [24]. Therefore, we aim to explore whether LPA_3_ signaling may constitute a new approach to maintain mitochondrial homeostasis and alleviate mitochondrial oxidative stress during the aging process. The outcomes of this investigation will advance our understanding of the molecular mechanisms leading to mitochondrial homeostasis in aging and novel intervention strategies against these pathological conditions.

## 2. Materials and Methods

### 2.1. Cell Culture and Pharmacological Reagents

Human cervical cancer HeLa and Human embryonic kidney HEK293 cells were obtained from ATCC and maintained in Dulbecco’s modified eagle medium (DMEM, Thermo Fisher Scientific, Waltham, MA, USA) supplemented with 10% fetal bovine serum (FBS, Thermo Fisher Scientific) and 1% penicillin/streptomycin (Pen/Strep, Lonza, Gampel-Bratsch, Switzerland). Human erythroleukemia K562 cells were obtained from ATCC and cultured in RPMI 1640 (Thermo Fisher Scientific) supplemented with 10% FBS and Pen/Strep. Hutchinson-Gilford progeria syndrome (HGPS) fibroblast lines AG11513F and age-matched control fibroblast line AG08470B were received from the Coriell Institute (Camden, NJ, USA) and were maintained in minimum essential medium Eagle-alpha modification (Alpha MEM, Thermo Fisher Scientific) supplemented with 15% FBS. Normal human skin fibroblasts (HSF, passage 9) were maintained in the same culture condition. HSF cells were derived from neonatal foreskin and used in our previous work [15]. Cells were cultured at 37 °C in a humidified atmosphere of 5% CO_2_.

For pharmacological treatment, cells were incubation with OMPT (Cayman Chemicals, Ann Arbor, MI, USA), hydrogen peroxide (H_2_O_2_), N-acetyl cysteine (NAC, Sigma, Burbank, CA, USA), Carbonyl cyanide p-trifluoro-methoxyphenyl hydrazone (FCCP, Sigma), or cisplatin (Thermo Fisher Scientific), for 4 to 48 h.

### 2.2. Plasmid Construct, siRNA and Transfection

The PCR products of the LMNA and progerin gene were cloned into a pAS_2_V_1_ vector, which used in our previous study [15]. The vectors were then transfected into HEK293 cells with Lipofectamine 2000 (Thermo Fisher Scientific) and selected by puromycin at 2 ug/mL for positive clone.

Small interfering RNA (siRNA) oligonucleotide duplexes designed against LPA_3_ [15], were custom synthesized (Thermo Fisher Scientific) and transfected with Lipofectamine 3000 (Thermo Fisher Scientific). Sequences for synthesis of siRNA were as follows: Control: 5′-GGU UAA GUC GCC CUC GCUC dTdT-3′, LPA_3_-1: 5′-CCA UUA AUC ACU GCU AGA UUU dTdT-3′; LPA_3_-2: 5′-CAG UAC AUA GAG GAU AGU AUU dTdT-3′. The gene accession number of the genes were as follows: lamin A (NM_170707.4), Progerin (NM_001282626.2), and LPA_3_ (NM_012152.3).

### 2.3. Western Blot

Whole cell lysate was prepared with lysis buffer (50 mM Tris pH = 7.5, 0.2 M NaCl, 1% Tween-20, 1% NP40, 1 mM sodium orthovanadate, 2 mM β-glycerophosphate and prote-ase inhibitors). Twenty μg of protein were seperated by 10% SDS polyacrylamide gel electrophoresis (PAGE) and transferred to pol-yvinylidene difluoride membranes (Merck, Darmstadt, Germany). Membranes were blocked by 5% BSA in Tris-buffered saline (20 mM Tris, pH 7.4, 150 mM NaCl) containing 0.1% Tween-20 (TBST) and incubated with primary antibodies overnight at 4 °C. Membranes were then reacted with horseradish peroxidase-conjugated secondary antibodies (Santa Cruz, Dallas, TX, USA) and imaged by X-ray films. Primary antibodies for western blot were as follows: anti-ANT1 (NBP83928, Novus, St Charles, MO, USA), anti-ANT2 (14671, Cell Signaling, Danvers, MA, USA), anti-GPX1 (3286S, Cell Signaling), anti-SOD1 (2770S, Cell Signaling), an-ti-SOD2 (13141S, Cell Signaling), anti-Actin (sc-8432, Santa Cruz), and anti-Ku70 (SC-17789, Santa Cruz).

### 2.4. Analysis of Cellular ROS and Mitochondrial Membrane Potential

For the detection of cellular ROS, exponentially growing cells were washed twice with PBS and then incubated with 20 μM 2′,7′ –dichlorofluorescin diacetate (DCFDA, Thermo Fisher Scientific) in a 37 °C incubator for 30 min. For the detection of mitochondria membrane potential (MMP, ΔΨm), exponentially growing cells were incubated with culture medium containing 200 nM of tetramethylrhodamine ethyl ester (TMRE, Thermo Fisher Scientific) in a 37 °C incubator for 30 min. All experiments were then detected by Amnis FlowSight imaging flow cytometry (Luminex, Austin, TX, USA), Keyence BZ-X700-All-in-one Fluorescence microscope (Keyence, Osaka, Japan) or Cytation 5 Multi-Mode Cell Imaging Reader (BioTek, Winooski, VT, USA). The mean fluorescent intensity analyzed in each treatment group was quantified and normalized to the control group.

### 2.5. Cell Proliferation Assay

1000 cells were plated in 96-well microplate overnight, followed by treatment with OXPHOS inhibitors, 0.01–1 μM antimycin (Sigma) or 0.01–1 μM rotenone (Sigma), for 3 days. Cells were then stained with DAPI and the cell number were analyzed using a Cytation 5 Multi-Mode Cell Imaging Reader (BioTek). The mean fluorescent intensity analyzed was quantified and normalized to the fluorescent intensity of DAPI and control group.

### 2.6. In Situ Proximity Ligation Assay (PLA) and Immunofluorescence Analysis 

Cells were harvested, followed by fixation with 4% PFA and permeabilization by 0.5% Triton X. in situ Proximity ligation assay (PLA) was performed by using Duolink PLA reagents (MilliporeSigma, Burlington, MA, USA) according to the manufacturer’s instructions. Cells were blocked with Duolink in situ blocking solution for 1 h before being probed with primary antibodies in Duolink in situ antibody diluent overnight at 4 °C. Cells were then incubated with oligonucleotides-conjugates secondary antibodies (PLA probe anti-rabbit PLUS and anti-mouse MINUS), followed by ligation and amplification with the fluorophore-labeled oligonucleotide probe (excitation = 495 nm, emission = 527 nm). For immunofluorescence staining, cells were incubated with primary antibodies overnight and then labeled with secondary antibodies for 1 h. Cells were stained with DAPI and the fluorescent images were developed by Keyence BZ-X700-All-in-one Fluorescence microscope (Keyence). Primary antibodies for PLA and immunofluorescent staining were as follows: mouse anti-VDAC1 (820701, BioLegend, San Diego, CA, USA), rabbit anti-IP3R1 (A302-158, Bethyl, Montgomery, TX, USA), mouse anti-Cytochrome *c* (612503, BioLegend), and rabbit anti-HSP60 (12165, CellSignaling).

### 2.7. ADP-ATP Exchange Assay

The ADP-ATP exchange assay in microplates was performed following previous studies [25,26] with modification. Cells were resuspended in Kreb medium (10 mM HEPES pH = 7.4, 145 mM NaCl, 5 mM KCl, 2.6 mM CaCl_2_, 5.6 mM glucose, 5 mM glutamate, and 5 mM malate) containing 1.1 μM MgGreen 5K+ (Thermo Fisher Scientific), 1 μM Na_3_VO_4_, 1 μM NaF, 1 μM β-glycerophosphate and protease inhibitors, followed by permeabilization by 375 μM digitonin. Cells were then loaded in 96-well solid black polystyrene microplate (Thermo Fisher Scientific). The fluorescence signal was read by BioTek Cytation 5 Multi-Mode Reader with the absorption/emission wavelength at 506/531 nm. After 10 min of reading the basal level of MgGreen 5K+ fluorescence, 5 mM of ADP was added to initiate ADP-ATP exchange. 5 mM EDTA were added at the end of the experiment for minimum fluorescence (F_min_), then recorded the maximum fluorescence (F_max_) by adding 10 mM MgCl_2_. [Mg^2+^]_free_ was calculated from the equation [Mg^2+^]_free_ = [K_d_(F − F_min_)/(F_max_ − F)] − 0.068 mM, while assuming a K_d_ of 0.9 mM for the complex of MgGreen and Mg^2+^. Then, the efflux of ATP ([ATP]_e_) was calculated with the following equation:

$$\left(\frac{{\left[{\mathrm{Mg}}^{2+}\right]}_{t}}{{\left[{\mathrm{Mg}}^{2+}\right]}_{\mathrm{free}}}-1-\frac{{\left[\mathrm{ADP}\right]}_{t}\;\left(t=0\right)+{\left[\mathrm{ATP}\right]}_{t}\;\left(t=0\right)}{K_{\mathrm{ADP}}+{\left[{\mathrm{Mg}}^{2+}\right]}_{\mathrm{free}}})/(\frac{1}{K_{\mathrm{ATP}}+\left[{\mathrm{Mg}}^{2+}\right]}-\frac{1}{K_{\mathrm{ADP}}+\left[{\mathrm{Mg}}^{2+}\right]}\right)$$

[Mg^2+^]_t_ is the concentration of total free Mg^2+^ contained in the reaction solution before adding ADP. [ADP]_t_ (t = 0) and [ATP]_t_ (t = 0) are the concentrations of ADP and ATP, respectively, at time zero, which is 0 for both. To calculate [ATP]_e_, we applied K_d_ values from references due to similar experimental conditions (K_ADP_ = 0.906 mM and K_ATP_ = 0.114 mM). 

### 2.8. Cellular Bioenergetics Measurements by Seahorse Assay 

To measure the rate of oxidative phosphorylation (OXPHOS), a Seahorse XFe24 Analyzer (Agilent Technologies, Santa Clara, CA, USA) was applied, according to manufacturer’s instructions. For measuring the oxygen consumption rate (OCR), 5 × 10^4^ cells were seeded in an XF assay plate overnight. Cells were incubated in a non-CO_2_ incubator at 37 °C for 1 h before experiments, and the culture medium was replaced with assay medium (medium without HEPES nor sodium bicarbonate). In addition, the cartridge was activated by calibration buffer overnight and calibrated by the XF24 analyzer before experiments. OCR was determined under basal conditions, then 0.25 μM oligomycin, 1 μM FCCP, and 1 μM rotenone plus antimycin A were added sequentially to estimate individual parameters of mitochondria.

### 2.9. Live Cell Imaging Analysis of Mitochondrial Calcium Concentration 

Cells were plated on Lab-Tek Chambered Coverglass (Thermo Fisher Scientific), and incubated in Krebs-Ringer-HEPES-glucose buffer (KRH-glc, 136 mM NaCl, 10 mM HEPES, 4.7 mM KCl, 1.25 mM MgSO_4_, 1.25 mM CaCl_2_, 25 mM glucose, pH7.4) containing 1 μM Rhod-2, AM (Cayman) and 0.002% Pluronic^®^ F-127 (Sigma) for 30 min at 37 °C incubator. After three times washed with KRH-glc, cells were further incubated at 37 °C for 30 min. Live cell images were preformed using Keyence BZ-X700-All-in-one Fluorescence microscope, equipped with Plan Apochromat 60× oil objective in warm 37 °C chamber. 1 mM ATP was used to stimulate P2Y receptors-dependent intracellular Ca^2+^ releasing from ER to mitochondria.

### 2.10. Statistical Analysis

Each result represents at least three independent experiments. The data are presented as mean ± SD. Differences between control and experimental groups were determined with *t*-test or one-way analysis of variance (ANOVA). Results with *p* * ≤ 0.05, *p* ** ≤ 0.01, *p* *** ≤ 0.001 were considered statistically significant. 

## 3. Results

### 3.1. LPA_3_ Signaling Is Crucial to Maintain Mitochondria Homeostasis and ROS Accumulation 

**Figure 1:**
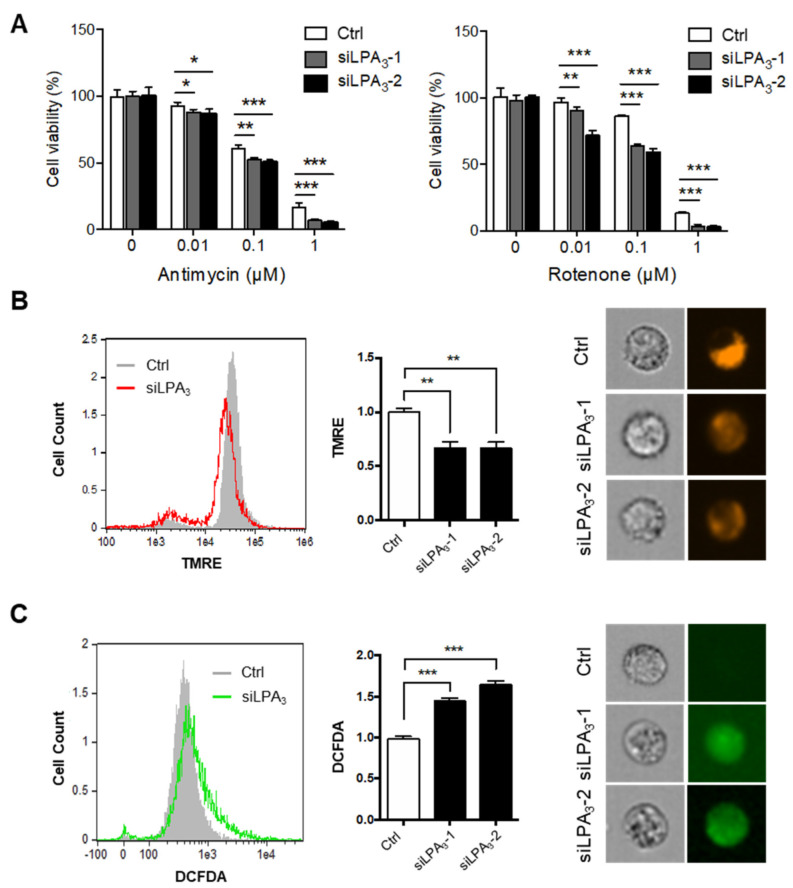
Impairment of mitochondrial homeostasis in LPA_3_–deficient cells. (**A**) Control (siRNA control) and two LPA_3_ siRNA knockdown HeLa cells were subjected to a cell proliferation assay against mitochondrial electron transport chain (ETC) inhibitors, complex-III inhibitor Antimycin A and complex-I inhibitor Rotenone, at indicated doses for 72 h. LPA_3_ siRNA knockdown cells were sensitive to inhibitors against ETC. (**B**) Mitochondrial ΔΨm of siRNA control and two LPA_3_ siRNA knockdown cells were analyzed with TMRE dye by imaging flow cytometry. TMRE fluorescence was reduced in two LPA_3_ siRNA knockdown cells. Representative images of TMRE stained cells were shown in the right panel. (**C**) Cellular ROS of siRNA control and two LPA_3_ siRNA knockdown cells were analyzed by DCFDA dye. Cells were stained with 20 μM of DCFDA at 37 °C for 30 min, followed by imaging flow cytometry analysis. DCFDA fluorescence showed increasing in two LPA_3_ siRNA knockdown cells. Representative images of DCFDA stained cells were shown in the right panel. The bar graphs were generated from three independent analyses. * *p* < 0.05; ** *p* < 0.01; *** *p* < 0.001.

To delineate the role of LPA_3_ and its underlying mechanism in regulating mitochondrial activity, we subjected the siRNA control and LPA_3_ siRNA-treated human cervical cancer HeLa cells to a cell proliferation assay with electron transport chain (ETC) complex I inhibitor, Rotenone, and complex III inhibitor, Antimycin A (Figure 1A). Two LPA_3_ siRNA were used in this study (Figure S1). LPA_3_ siRNA knockdown cells were susceptible to the two ETC inhibitors, suggesting that that LPA_3_ was required to counter mitochondrial stress. Furthermore, the mitochondrial membrane potential (ΔΨm) was measured by tetramethylrhodamine ethyl ester, perchlorate (TMRE) staining. TMRE is a cell-permeant that shows the level of negative charge across the healthy inner mitochondrial membrane to the matrix. ETC complex I, III, and IV serve as proton pumps to generate a proton gradient (ΔΨm) across the inner mitochondrial membrane as energy storage for driving OXPHOS. Thus, mitochondrial ΔΨm is a remarkable indicator for mitochondrial healthiness. We found significantly depolarized mitochondrial ΔΨm in LPA_3_-depleted cells (Figure 1B). Since superoxide can leak out from mitochondria to contribute endogenous ROS, the cellular ROS was detected by 2′,7′–dichlorofluorescein diacetate (DCFDA) staining. It showed that the cellular ROS accumulation increased in LPA_3_-depleted cells (Figure 1C).

**Figure 2:**
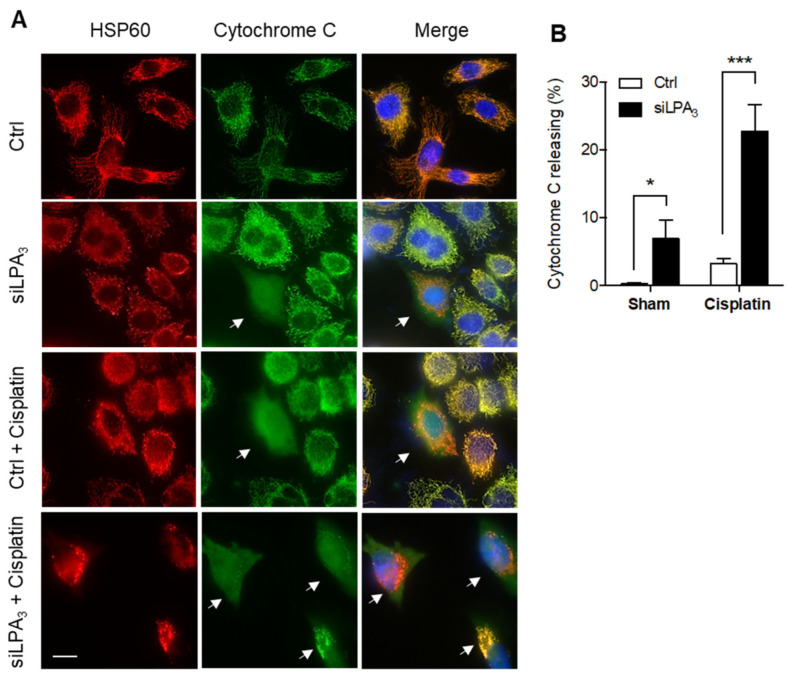
Knockdown of LPA_3_ increased the release of cytochrome *c* from mitochondria to the cytosol. (**A**) Representative images of immunofluorescent staining that showed cytochrome *c* (green) and mitochondria marker HSP60 (red) in siRNA control (Ctrl) and LPA_3_ siRNA knockdown (siLPA_3_) HeLa cells. Nuclei were stained with DAPI. Cytochrome *c* releasing cells were indicated with the white arrow. Scale bar represents 10 µm. (**B**) Knockdown of LPA_3_ increased the release of cytochrome *c* from mitochondria to the cytosol and enhanced cellular sensitivity to 0.5 μM of cisplatin-induced cytochrome *c* releasing. The bar graphs were generated from three independent analyses. * *p* < 0.05; *** *p* < 0.001.

Moreover, cytochrome *c* release was monitored to detect the healthiness of mitochondria. In healthy mitochondria, cytochrome *c* locates at the mitochondrial intermembrane space and functions as an electron shuttle in the ETC. The mitochondrial damage induces the outer membrane’s permeabilization, facilitating cytochrome *c* releasing from the mitochondria to cytosol. The released cytochrome *c* in the cytosol further mediates the activation of caspase-3 dependent apoptosis [27]. LPA_3_ siRNA knockdown cells displayed an increased ratio of cytochrome *c* release. Moreover, cisplatin as a mitochondrial DNA damaging reagent was treated to induce cytochrome *c* releasing [28]. Relative to siRNA control HeLa cells, LPA_3_ depletion cells were more sensitive to cisplatin-induced cytochrome *c* releasing (Figure 2). These results highlight the crucial role of LPA_3_ in mitochondria homeostasis, evidenced by the cytochrome *c* releasing, mitochondrial ΔΨm depolarization, and cellular ROS accumulation in LPA_3_-depletion cells.

### 3.2. LPA_3_ Signaling Is Involved in Mitochondrial ADP-ATP Exchange

**Figure 3:**
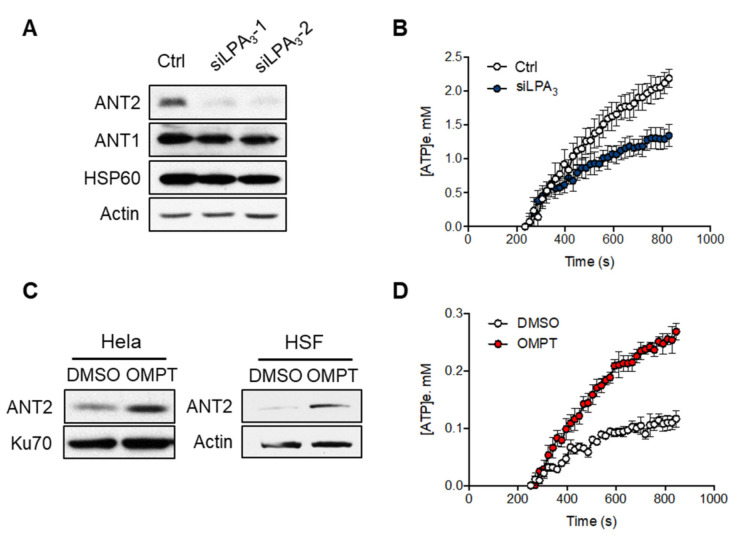
LPA_3_ modulated mitochondrial ADP-ATP exchange activity. (**A**) Representative western blot analysis of the attenuation of ANT2 protein level, but not ANT1, in LPA_3_ knockdown cells. Actin was used as a loading control. (**B**) siRNA control (Ctrl) and LPA_3_ siRNA knockdown (siLPA_3_) HeLa cells were subjected to ADP-ATP exchange assay using MgGreen 5K+ fluorescent dye. The exchange was stimulated by 5 mM of ADP. MgGreen 5K+ fluorescence was first measured to indicate changes in free magnesium concentration after adding ADP. The decreased free magnesium concentration was then converted to concentration of efflux ATP ([ATP]e, mM). Treatment of LPA_3_ siRNA attenuated the ADP-ATP exchange. (**C**) Representative western blot analysis of the induction of ANT2 protein level by treatment of 50 nM of LPA_3_ agonist OMPT for 48 h in HeLa and Human skin fibroblast (HSF). (**D**) Treatment with 50 nM of OMPT increased the ADP-ATP exchange. The graphs were generated from three independent analyses.

Previous research has shown that low expression of ANT2 is linked to mitochondrialinherent to cell senescence [29]. Therefore, we aimed to test if LPA_3_ signaling regulates protein level and activity of ANT2. The Western blot analysis of mitochondrial proteins showed that the protein level of ANT2 was significantly reduced by siLPA_3_, whereas ANT1 and the mitochondrial matrix chaperone HSP60 were not (Figure 3A). Adenine nucleotide translocase 2 (ANT2) is the crucial regulator for mitochondrial homeostasis by exchanging ADP and ATP across the mitochondrial inner membrane. In most physiological conditions, electron transport through the ETC is tightly coupled with the phosphorylation of ADP to ATP by F0F1-ATPase. Impairing the ADP supply to mitochondria will slow down OXPHOS, increase the redox status of mitochondria, and thereby drive excess ROS production [30]. Thus, we measured the mitochondrial ADP-ATP exchange using an Mg^2+^ sensitive fluorescent probe Magnesium Green 5K+ (abbreviated as MgGreen) [25,26]. Since biologically active ATP and ADP are bound with Mg^2+^ and have different affinity (K_ADP_ = 0.906 mM and K_ATP_ = 0.114 mM). The dynamics of free extra-mitochondrial Mg^2+^ can be measured and determine ADP-ATP exchange activity in cells. We observed that the LPA_3_ knockdown cells by siRNA showed a significant attenuation in ADP-ATP exchange (Figure 3B). Moreover, activation of LPA_3_ with its highly selective agonist, OMPT, increased the ANT2 protein expression in both Hela and human skin fibroblast (HSF) cells (Figure 3C) and the activity of ADP-ATP exchange (Figure 3D). These results suggest that LPA_3_ signaling is crucial to regulate the mitochondrial ADP-ATP exchange.

### 3.3. LPA_3_ Signaling Is Involved in the Regulation of Mitochondrial Ca^2+^ Influx from ER to Mitochondria

**Figure 4:**
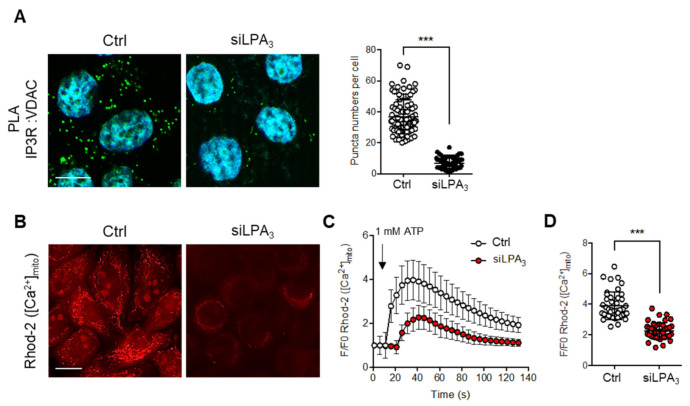
LPA_3_ ablation reduced IP3R1 and VDAC1 association and disrupts mitochondria Ca^2+^ influx. (**A**) Representative images of in situ proximity ligation assay were used to unveil the IP3R1-VDAC1 interaction in siRNA control (Ctrl) and LPA_3_ siRNA knockdown (siLPA_3_) HeLa cells. Green dots indicated the location of the interaction. Nuclei were stained with DAPI. The association between VDAC1 and IP3R1 was significantly attenuated in LPA_3_ knockdown cells. Scale bar represents 10 µm. (**B**) Representative images of mitochondrial [Ca^2+^] analysis by mitochondria-specific Ca^2+^ indicator Rhod-2 AM. Mitochondria [Ca^2+^] were measured in siRNA control (Ctrl) and LPA_3_ siRNA knockdown (siLPA_3_) HeLa cells. Scale bar represents 10 µm. (**C**) Representative traces of intramitochondrial Ca^2+^ dynamic. 1 mM of ATP was used to stimulate P2Y receptors-dependent intracellular Ca^2+^ releasing from ER. (**D**) The average Rhod-2 AM peak fluorescence following administration of ATP was decreased in LPA_3_ knockdown cells. The graphs were generated from three independent analyses. *** *p* < 0.001.

On the other hand, the mitochondrial electrochemical proton gradient (ΔμH^+^) is the major component of the mitochondria ΔΨ. ΔμH^+^ drives the flow of H^+^ through ATP synthase in a reaction coupled to the generation of ATP from ADP [31] and represents a substantial driving force for Ca^2+^ accumulation [32]. Mitochondrial Ca^2+^ traffics from ER to mitochondria in response to physiological stimuli and stresses [33,34]. The protein channel complex IP3R1-VDAC1 was suggested to transfer the mitochondrial Ca^2+^ influx from the ER to mitochondrial intermembrane space. Therefore, the interacting structure of IP3R1-VDAC1 was detected with in situ proximity ligation assay (PLA) by probing IP3R1 and VDAC1 antibodies. The hybridization with PLA secondary antibodies coupled to fluorescent oligonucleotides hybridizes if the distance is <40 nm [35]. The interactions between IP3R1-VDAC1 were decreased in LPA_3_ depleted cells (Figure 4A). To verify the Ca^2+^ transport efficiency from the ER to mitochondria, the mitochondrial Ca^2+^ was measured by mitochondria-specific Ca^2+^ indicator Rhod-2 AM with live cell image analysis. ATP was used to stimulate P2Y receptors-dependent intracellular Ca^2+^ releasing from the ER. By measuring the dynamics and the peaks of Rhod-2 AM fluorescence, we demonstrated that the rate and amount of Ca^2+^ efflux into mitochondria following administration of ATP treatment were significantly attenuated in LPA_3_ depleted cells (Figure 4B–D). The results highlight that LPA_3_ signaling is important to support the structure of the Ca^2+^ channel complex and Ca^2+^ trafficking into the mitochondria from ER.

### 3.4. LPA_3_ Signaling Participates in the Regulation of Mitochondrial Stress Response

**Figure 5:**
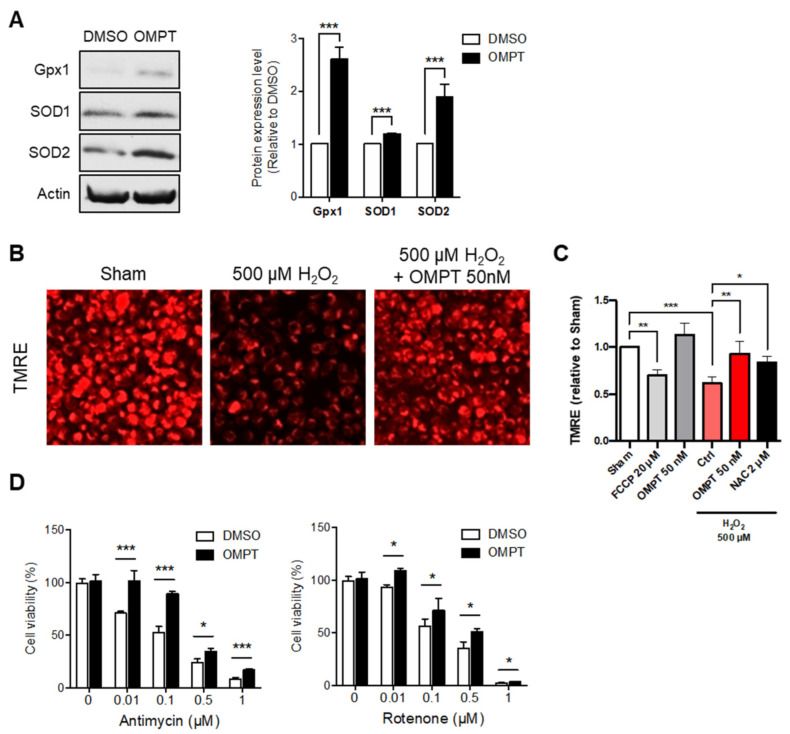
LPA_3_ protected against oxidative stress-induced mitochondria dysfunction. (**A**) Western blot results show that activating LPA_3_ by 50 nM LPA_3_ agonist, OMPT for 7 h enhanced protein levels of Gpx1, SOD1, and SOD2 in HeLa cell. Actin was used as a loading control. (**B**) Representative images of mitochondrial membrane potential (ΔΨm) analysis by TMRE staining. Scale bar represents 10 µm. (**C**) Cells were pre-treated 50 nM of OMPT or 2 μM of NAC for 4 h respectively, followed by 3 h 500 μM of H_2_O_2_ treatment. Cells were further stained with 200 nM of TMRE and 1 μM of DAPI for 30 min at 37 °C, followed by Imaging Plate Reader analysis. The fluorescent intensity of DAPI was used as cell number control. HeLa cells showed decreased mitochondria ΔΨm under 500 μM of H_2_O_2_ treatment. 50 nM of OMPT and 2 μM of NAC protected cells against H_2_O_2_-induced mitochondrial ΔΨm loss. FCCP was used as a positive control for loss of mitochondrial ΔΨm. (**D**) HeLa cells were subjected to a cell proliferation assay against mitochondrial electron transport chain (ETC) inhibitors, complex-III inhibitor Antimycin A (left), and complex-I inhibitor Rotenone (right), at indicated doses for 72 h. Cells showed protective effects against ETC inhibitors in 50 nM of OMPT treatment. The bar graphs were generated from three independent analyses. * *p* < 0.05; ** *p* < 0.01; *** *p* < 0.001.

Since we have shown that LPA_3_ plays a crucial role as regulator of mitochondria, we next aimed to discuss whether LPA_3_ signaling may constitute a new approach against oxidative stress-induced mitochondrial dysfunction. Activation of LPA_3_ with its highly selective agonist, OMPT, showed potent induction of mitochondrial antioxidants, glutathione peroxidase 1 (Gpx1), and superoxide dismutase type 1 and type 2 (SOD1 and SOD2) protein expression in human cervical cancer HeLa cells (Figure 5A). To further delineate the role of LPA_3_ in regulating the mitochondrial oxidative stress response, we subjected HeLa cells with treatment of hydrogen peroxide (H_2_O_2_), followed by analysis of the mitochondrial membrane potential (ΔΨm) by TMRE staining. We found significantly depolarized mitochondrial ΔΨm in H_2_O_2_-induced oxidative stress and rescued with OMPT treatment (Figure 5B,C). The mitochondrial uncoupler carbonyl cyanide *p*-trifluoromethoxyphenylhydrazone (FCCP) was used as a negative control to depolarize mitochondria. The antioxidant N-acetylcysteine (NAC) was used as the positive control to rescue ΔΨm. Human erythroleukemic K562 cells were also used to confirm the protection effect of OMPT (Figure S2). The results also suggested similar antioxidant function of OMPT. In addition, we further subjected HeLa cells to a cell proliferation assay with inhibitors against OXPHOS. The respiratory complex I (NADH: ubiquinone oxidoreductase) and respiratory complex III (ubiquinone: cytochrome *c* reductase) are recognized to produce mtROS relevant to pathological aged diseases [36]. While highly mtROS is presented, the respiratory complexes are likely to be attacked and drive reverse electron transfer (RET), which at the same time leads to electron leak to damage cell compartments [37]. The OMPT treated cells were highly resistant to ETC inhibitors Rotenone (complex I) and Antimycin A (complex III) induced oxidative stress (Figure 5D). These findings suggested the protective functions of LPA_3_ to alleviate mitochondrial function during oxidative stress.

### 3.5. LPA_3_ Activation Rescues Mitochondrial Activity in Hutchinson-Gilford Progeria Syndrome Cells

**Figure 6:**
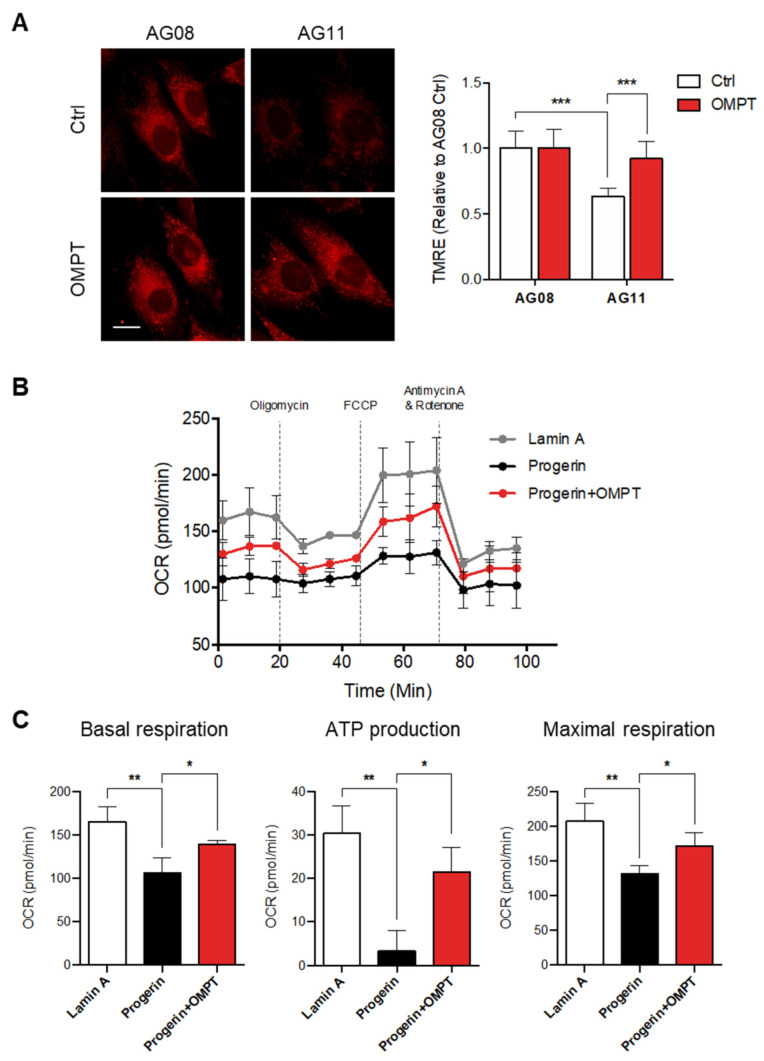
Activation of LPA_3_ rescued mitochondrial activity in Hutchinson-Gilford progeria syndrome cells. (**A**) HGPS patient fibroblast AG11 and age-matched control fibroblast line AG08 cells were treated with 50 nM of OMPT or DMSO control for 48 h. Cells were further stained with 200 nM of TMRE for 30 min at 37 °C, followed by Imaging analysis. Analysis of mitochondrial ΔΨm by TMRE staining showed decreased mitochondrial ΔΨm in HGPS AG11 cells relative to the AG08 control cells. OMPT increased the mitochondrial ΔΨm in HGPS AG11, but not AG08 control cells. Representative images of TMRE stained cells were shown in the left panel. Scale bar represents 10 µm. (**B**) Lamin A control and HGPS progerin expression HEK 293 cells were subjected to Agilent Seahorse XFe24 and analyzed to measure oxidative phosphorylation (OXPHOS) by measuring the oxygen consumption rate (OCR). (**C**) HGPS cells displayed a reduction of OCR, basal respiration, ATP production, and maximal respiration and were rescued by 50 nM of OMPT treatment. The bar graphs were generated from three independent analyses. * *p* < 0.05; ** *p* < 0.01; *** *p* < 0.001.

The ROS leads to cumulative damage during the aging process and results in irreversible cell damage. The mitochondria are considered the central regulators of redox activities, and oxidative stress is referred to as mitochondrial ROS (mtROS). Therefore, the alterations in mitochondrial functions have been suggested to be the hallmark and the drivers of aging. Our previous publication indicated that the expression level of LPA_3_ declined in the aging disease Hutchinson-Gilford progeria syndrome (HGPS). Enhancement of LPA_3_ signaling by agonist is suggested to ameliorate premature aging [11,15]. HGPS also displayed the dysregulation of OXPHOS and mitochondrial activity [22]. Therefore, we aimed to explore whether the protective effects of LPA_3_ on mitochondria and against oxidative stress can constitute a new approach to HGPS treatment. We first showed that OMPT treatment protected normal fibroblast against H_2_O_2_ induced oxidative stress (Figure S3). Next, we analyzed the mitochondrial ΔΨm in HGPS patient fibroblast AG11 and age-matched control fibroblast line AG08 cells by TMRE staining. Relative to the control AG08 cells, HGPS AG11 cells displayed depolarized mitochondrial ΔΨm. Activation of LPA_3_ signaling had no effect on control cells but rescued depolarized mitochondrial ΔΨm in HGPS cells (Figure 6A). Moreover, we subjected lamin A control and HGPS progerin expression HEK 293 cells to the Agilent Seahorse XFe24 analyzer to determine mitochondrial OXPHOS by measuring the oxygen consumption rate (OCR). The OCR was determined under basal conditions followed by the addition of oligomycin (ATP synthase inhibitor), FCCP (mitochondrial uncoupler), and rotenone plus antimycin A (respiratory complex I and III inhibitors) to estimate individual parameters of mitochondria. Seahorse XF Cell Mito Stress assays revealed that the OXPHOS was significantly disrupted in HGPS cells (Figure 6B,C). OMPT treatment rescued the OCR level of basal respiration, ATP production, and maximal respiration (Figure 6B,C). Together, we conclude that LPA_3_ acts as the upstream signal to maintain mitochondria homeostasis through regulating mitochondria Ca^2+^ trafficking and ADP-ATP exchange. Loss of LPA_3_ leads to dysregulation of mitochondria biogenesis, OXPHOS, and ROS control in HGPS and aging cells. Activating LPA_3_ signaling by its agonist OMPT can rescue the mitochondrial defects from these pathological conditions. These results suggested that the potentiation of LPA_3_ signaling may constitute a new approach against aging progress and mitochondrial oxidative stress.

## 4. Discussion

Mitochondria are considered as both the source and the target of ROS. ROS, such as superoxide and hydrogen peroxide, are the by-products of mitochondrial aerobic metabolism. Two different sites in the ETC, respiratory complex I (NADH dehydrogenase) and respiratory complex III (ubiquinone-cytochrome *c* reductase), are recognized as the primary sites of ROS production [38]. Under normal conditions, ROS are controlled at the physiological levels by several endogenous antioxidants and scavengers, including SODs, catalase, and glutathione peroxidase. Normal levels of ROS are important physiological regulators of the cellular signaling pathway [39]. However, down-regulation or defects in antioxidant enzymatic systems in the aging process contribute to the accumulation of high ROS (hiROS) [40]. When hiROS is presented, the respiratory complexes are likely to be attacked and drive reverse electron transfer (RET), which at the same time leads to electron leak to damaged cell compartments. Consequently, the OXPHOS activities will be prohibited to lower the production of ROS, whereas ATP production from mitochondria will also be reduced. On the other hand, mitochondrial membrane potential (MMP, ΔΨm) established by the proton gradient through ETC is essential to drive both F0F1-ATPase to produce ATP and acts as quality control for mitochondrial maintenance. Due to the reduction of ATP production and lower mitochondrial ΔΨm, the accumulated ROS drives the cell senescence [41]. Moreover, the ROS-induced oxidative stress further leads to modification of cellular proteins, lipids, and DNA that are generally more irreversible [42]. Therefore, the interplay between mitochondria ROS and protective antioxidant responses is essential for aging.

Mitochondrial defects and the perturbation of endoplasmic reticulum–mitochondria contact sites (MERC) are indicated as a hallmark in aging and many aging-associated neuronal degeneration diseases, including Alzheimer’s disease (AD), Parkinson’s disease, and amyotrophic lateral sclerosis [43,44,45]. The communication between ER and mitochondria regulates lipid metabolism, Ca^2+^ homeostasis, unfolded protein response, ER stress, and mitochondrial quality control, all implicated in neurodegenerative diseases [46]. Furthermore, Studies unveil that the cerebral metabolic rate of oxygen is significantly decreased and correlated to the severity of dementia in the brain of AD patients [47]. Mitochondria are the primary source for energy generation in neuron cells through OXPHOS, which involves a flow of electrons through ETC and consumes oxygen. Therefore, reduced glucose usage, so-called “glucose hypo-metabolism,” strongly implies mitochondrial dysfunction in the course of AD [48]. In addition, the failure of ETC leads to RET and constitutes a positive feedback loop for enhancing ROS production, inducing mitochondria dysfunction, and releasing more ROS. Excessive generation of ROS will harm all compartments of cells which drive cell death signaling. This study indicates that the reduction of MERC Ca^2+^ IP3R-VDAC channel, and ANT2 activity in LPA_3_-deficient cells impairs the import of Ca^2+^ and ADP into the mitochondrial matrix. These limit the efficiency of F0F1-ATPase in the final step of the OXPHOS reaction and consequently attenuate electron transfer through the ETC and increase the production of toxic ROS by RET. Notably, dysfunction of LPA pathways is also reported in AD [49]. Thus, we suspect that the absence of the LPA_3_ leads to the dysregulation of MERC and ANT2 during progression of neuronal degeneration diseases. Since LPA_3_ has a beneficial effect on mitochondrial health and protective antioxidant responses, the LPA_3_ agonists hold value as potential drugs against the pathogenesis of neuronal degeneration diseases in the future.

On the other hand, LPA_3_ has been reported to protect erythroid differentiation and aging-related anemia [11,50]. The regulation of mitochondrial biogenesis is also important in erythropoiesis since mitochondria is crucial for heme and iron metabolism. Extracellular Ferric iron (Fe^3+^) binds to a carrier protein transferrin (Tf) and is transported into the cell by Tf receptor-mediated endocytosis [51]. A proton pump then acidifies the iron-loaded endosomes to change the conformation of the Tf and Tf receptor, followed by iron releasing [52]. Ferric iron is converted into ferrous iron (Fe^2+^) in the endosomes. These released iron ions are transferred across the endosomal membrane to the cytoplasm by divalent metal transporter 1 (DMT1) [53]. Next, intracellular iron is further transported to mitochondria for heme synthesis and iron-sulfur cluster (Fe-S) biogenesis. In addition, heme and iron-sulfur clusters are crucial for erythropoiesis and function as an important cofactor of enzymes for various cellular physiologies [54]. Therefore, defective mitochondria could seriously impact erythropoiesis and systemic defect. Moreover, impaired iron metabolism will lead to iron overload in multiple organs, which is called hemochromatosis. Iron release and overload from damaged erythrocytes cause cell damage through ferroptosis, a recently identified iron-dependent form of programmed cell death [55]. The high redox potential of iron leads to the Fenton reaction, leading to accumulation of toxic oxidative stress, lipid peroxidation in the plasma membrane, and various organelle damage. The metabolic rate decreases with aging, leading to deficiency in molecules using iron as cofactors or working on iron metabolism [56]. Accordingly, iron would be in excess with aging progression, leading systemic ferroptosis. These findings align with our results and suggest that LPA_3_ may provide beneficial effects against aging/anemia-induced ferroptosis in multiple organs.

## 5. Conclusions

This study demonstrated that the LPA_3_ signal is crucial for mitochondrial biogenesis and oxidative stress response. siRNA-mediated depletion of LPA_3_ led to the depolarization of mitochondrial ΔΨm and ROS accumulation. In addition, depletion of LPA_3_ by siRNA resulted in cisplatin-induced cytochrome *c* releasing, suggesting that LPA_3_ is essential to suppress the mitochondrial apoptosis pathway. Moreover, LPA_3_ was associated with the ANT2 protein expression and ADP-ATP exchange activities. Depletion of LPA_3_ led to the destabilization of ANT2 and disrupted ADP-ATP exchange activities. In addition, LPA_3_ knockdown cells showed decreased calcium uptake from ER to mitochondria via the IP3R1-VDAC1 channel, which contributes to increasing ROS accumulation and mitochondria dysfunction. Notably, the LPA_3_ agonist OMPT protected mitochondrial functions against oxidative stress by H_2_O_2_ and OXPHOS inhibitors. Moreover, treatment of OMPT rescued mitochondrial activities in HGPS premature aging cells. Based on our findings, we propose that LPA_3_ acts as the gatekeeper for mitochondrial healthiness and protects cells from oxidative stress to maintain cell youth. Additionally, LPA_3_ agonists can be a promising therapeutic target to prevent mitochondrial oxidative stress in aged cells.

## Data Availability

The data is contained within the article.

## Supplementary Materials

The following supporting information can be downloaded at: https://www.mdpi.com/article/10.3390/antiox11020351/s1, Figure S1: The knockdown efficiency of LPA_3_ siRNA. LPA_3_ mRNA expression level was measured using real-time PCR and relative to siRNA control. The expression level of GAPDH was used as a loading control. The bar graph was generated from three independent analyses. *** *p* < 0.001. Figure S2: LPA_3_ protected against oxidative stress-induced mitochondrial ΔΨm loss in K562 cells. K562 cells showed decreased mitochondria ΔΨm under 500 μM of H_2_O_2_ treatment. 50 nM of OMPT and 2 μM of NAC protected cells against H_2_O_2_-induced mitochondrial ΔΨm loss. FCCP was used as a positive control for loss of mitochondrial ΔΨm. The bar graphs were generated from three independent analyses. *, *p* < 0.05; **, *p* < 0.01; ***, *p* < 0.001. Figure S3: LPA_3_ improved cell viability from H_2_O_2_ induced oxidative stress. HSF cells were subjected to a cell proliferation assay under 50 μM of H_2_O_2_ for 72 h. 50 nM of OMPT showed protective effects against H_2_O_2_. The bar graphs were generated from three independent analyses. **, *p* < 0.01.
