# Supplementary material for: Lysophosphatidic Acid Receptor 3 Promotes Mitochondrial Homeostasis against Oxidative Stress: Potential Therapeutic Approaches for Hutchinson–Gilford Progeria Syndrome
Source: Antioxidants (Basel). 2022 Feb 10;11(2):351. doi: 10.3390/antiox11020351 (PMC8869156; doi:10.3390/antiox11020351)
Supplement: Supplementary file 1 [file antioxidants-11-00351-s001.zip › antioxidants-1555712-supplementary.pdf]

Supplementary information for

Lysophosphatidic Acid Receptor 3 Promotes Mitochondrial Homeostasis against Oxidative Stress: Potential Therapeutic Approaches for Hutchinson–Gilford Progeria Syndrome

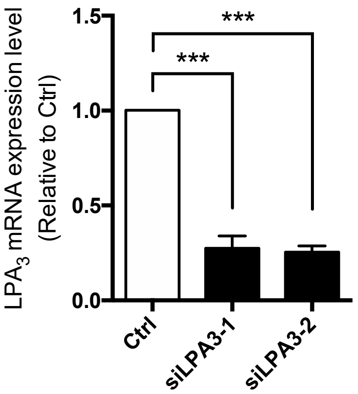

Figure S1. The knockdown efficiency of LPA_3 siRNA. LPA_3 mRNA expression level was measured using real-time PCR and relative to siRNA control. The expression level of GAPDH was used as a loading control. The bar graph was generated from three independent analyses. ***, P < 0.001.

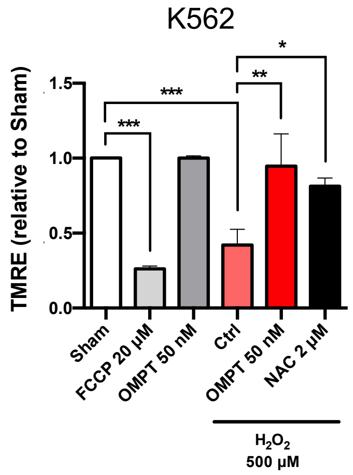

Figure S2. LPA_3 protected against oxidative stress-induced mitochondrial ΔΨm loss in K562 cells**.** K562 cells showed decreased mitochondria ΔΨm under 500 μM of H_2O_2 treatment. 50 nM of OMPT and 2 μM of NAC protected cells against H_2O_2-induced mitochondrial ΔΨm loss. FCCP was used as a positive control for loss of mitochondrial ΔΨm. The bar graphs were generated from three independent analyses. *, P < 0.05; **, P < 0.01; ***, P < 0.001.

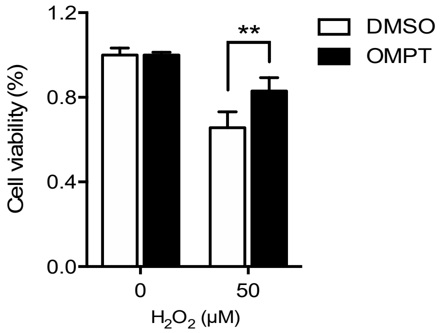

Figure S3. LPA_3 improved cell viability from H_2O_2 induced oxidative stress. HSF cells were subjected to a cell proliferation assay under 50 μM of H_2O_2 for 72 hr. 50 nM of OMPT showed protective effects against H_2O_2. The bar graphs were generated from three independent analyses. **, P < 0.01.
